# Germline IgM predicts T cell immunity to *Pneumocystis*

**DOI:** 10.1172/jci.insight.161450

**Published:** 2022-09-08

**Authors:** Kristin Noell, Guixiang Dai, Dora Pungan, Anna Ebacher, Janet E. McCombs, Samuel J. Landry, Jay K. Kolls

**Affiliations:** 1 Departments of Pediatrics and Medicine, Center for Translational Research in Infection and Inflammation, and; 2 Department of Biochemistry and Molecular Biology, Tulane University School of Medicine, New Orleans, Louisiana, USA.

**Keywords:** Immunology, Infectious disease, Adaptive immunity, Fungal infections, Proteomics

## Abstract

*Pneumocystis* is the most common fungal pulmonary infection in children under the age of 5 years. In children with primary immunodeficiency, *Pneumocystis* often presents at 3–6 months of age, a time period that coincides with the nadir of maternal IgG and when IgM is the dominant Ig isotype. Because B cells are the dominant antigen-presenting cells for *Pneumocystis*, we hypothesized the presence of fungal-specific IgMs in humans and mice and that these IgM specificities would predict T cell antigens. We detected fungal-specific IgMs in human and mouse sera and utilized immunoprecipitation to determine whether any antigens were similar across donors. We then assessed T cell responses to these antigens and found anti-*Pneumocystis* IgM in WT mice, *Aicda^–/–^* mice, and in human cord blood. Immunoprecipitation of *Pneumocystis murina* with human cord blood identified shared antigens among these donors. Using class II MHC binding prediction, we designed peptides with these antigens and identified robust peptide-specific lung T cell responses after *P*. *murina* infection. After mice were immunized with 2 of the antigens, adoptive transfer of vaccine-elicited CD4^+^ T cells showed effector activity, suggesting that these antigens contain protective *Pneumocystis* epitopes. These data support the notion that germline-encoded IgM B cell receptors are critical in antigen presentation and T cell priming in early *Pneumocystis* infection.

## Introduction

Due to the inability to culture *Pneumocystis spp*., data on incidence and prevalence of *Pneumocystis* infection is highly skewed toward Western populations and patients with severe pneumonia requiring hospitalization (1). However, recent epidemiology suggests that *Pneumocystis* is the most prevalent pulmonary fungal infection in the first year of life (2, 3), and the recent Pneumonia Etiology Research for Child Health (PERCH) study showed it was in the top 10 etiologies of childhood pneumonia in Mali, Kenya, and Zambia (4). *Pneumocystis pneumonia* requiring hospitalization in the first year of life in the US is highly associated with primary immunodeficiencies (PIDs) or immunodeficient states due to chemotherapy or high-dose steroids (5, 6). Among PIDs, we have recently shown that the murine model of *Pneumocystis murina* infection can recapitulate *Pneumocystis pneumonia* susceptibility of many PID syndromes, including severe combined immunodeficiency as well as IL-21R mutations (6).

Although dendritic cells play key roles in antigen (Ag) presentation in the lung, in the context of *Pneumocystis pneumonia*, B cells play critical roles as Ag-presenting cells. Depletion of B cells with anti-CD20 (7) or deletion of class II MHC expression on B cells (8–10) abrogates T cell priming and results in the failure of the host to clear *Pneumocystis* in the murine model. In the setting of PID, children rarely develop *Pneumocystis pneumonia* earlier than 3 months of age, likely due to protective effects of maternal IgG. Additionally, in the PERCH cohort, *Pneumocystis* was often diagnosed in children under the age of 1.5 years (4). Based on this, we hypothesized that germline-encoded IgM B cells are likely critical early Ag-presenting cells in infant *Pneumocystis pneumonia*, as this would be the dominant B cell repertoire at this age. If this were true, we should be able to detect anti-*Pneumocystis*–specific IgMs in naive mouse sera and human cord blood. Moreover, fungal proteins recognized by IgM should prime T cell responses in vivo. To test this, we examined IgM specificities in naive mouse sera and human cord blood and identified Ags recognized by human cord blood using proteomics. We went on to demonstrate that 2 of these Ags prime protective CD4^+^ T cell responses in vivo in a murine model of *Pneumocystis pneumonia*. Taken together, these data suggest that the IgM repertoire can be used to identify CD4^+^ T cell Ags to *Pneumocystis* that may aid in diagnosis as well as more objectively assessing need for prophylaxis.

## Results

### Baseline anti-Pneumocystis IgM titers in C57BL/6 mice.

**Figure 1:**
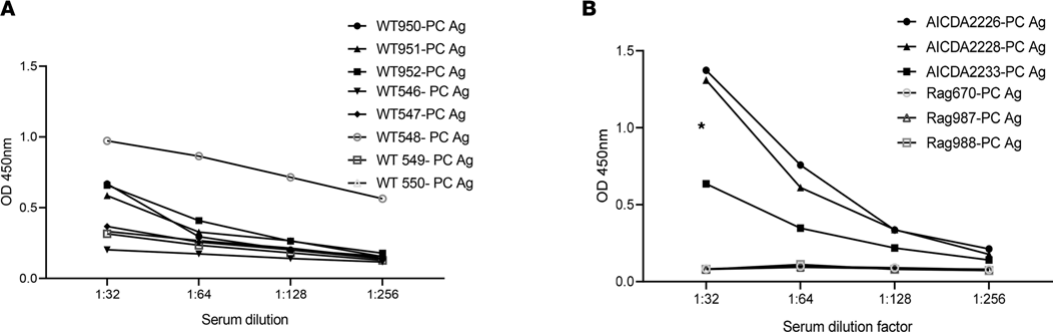
IgM responses to whole *Pneumocystis* antigen in *Pneumocystis*-naive mice. (**A**) Anti-*Pneumocystis* IgM titers in WT C57BL/6 mice. (**B**) Anti-*Pneumocystis* IgM titers in *Aicda^–/–^* and *Rag2^–/–^* mice. In this assay, an OD of more than 0.1 was considered positive. The asterisk denotes significant differences between *Aicda^–/–^* and *Rag2^–/–^* mice (by Mann-Whitney).

Previous work has shown that conserved natural IgM antibodies with carbohydrate specificities can mediate innate and adaptive immunity against *Pneumocystis* (11, 12). Given that *Pneumocystis* clearance is mediated by class II–restricted CD4^+^ T cells, we hypothesized that IgM with protein specificities may predict subsequent class II–dependent Ags. ELISA analysis of serum IgM in specific pathogen–free C57BL/6 mice showed detectable titers (OD > 0.1) of anti-*Pneumocystis* Ag (PC Ag) (Figure 1A). Based on our hypothesis, we predicted that similar results would be observed in mice deficient in cytosine deaminase (*Aicda^–/–^* mice), the enzyme that mediates class-switch recombination and somatic hypermutation. We detected significant anti-*Pneumocystis* IgM in *Aicda^–/–^* mice but not in *Rag2^–/–^* mice (Figure 1B), which also demonstrated the low background of the assay.

### Baseline anti-Pneumocystis IgM titers in human cord blood.

**Figure 2:**
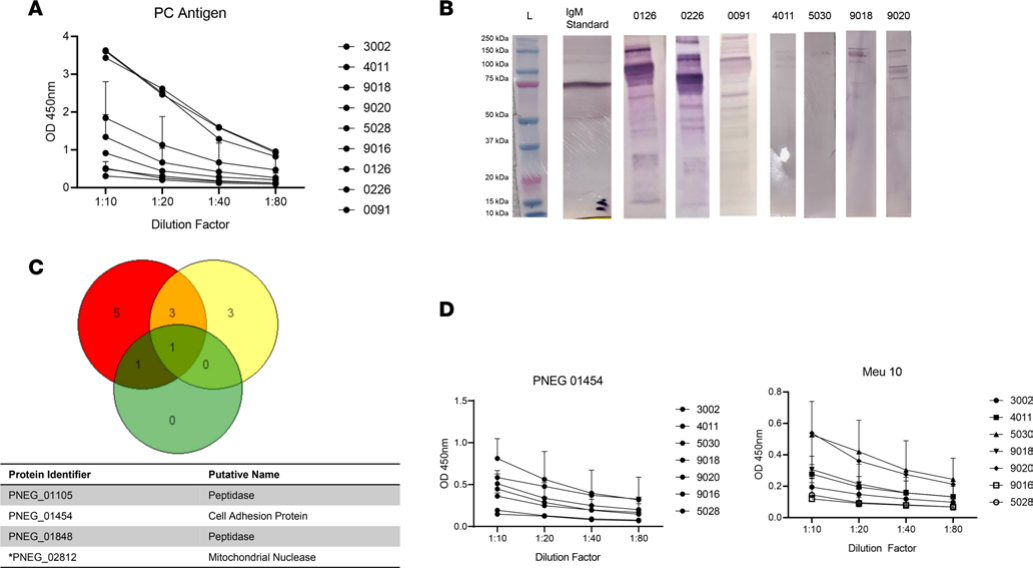
IgM responses to *Pneumocystis* antigens in human cord blood. (**A**) Anti-*Pneumocystis* IgM titers in human cord blood in response to whole *Pneumocystis* antigen. (**B**). Western blot analysis of human cord blood IgM in response to whole *Pneumocystis* antigen. (**C**). Venn diagram analysis of *Pneumocystis* proteins immunoprecipitated by human cord blood IgM. (**D**) Human cord blood IgM specificity in response to *Pneumocystis-*specific antigens PNEG_01454 and Meu10 (PNEG_01435). PNEG_02812 was identified in all 3 donors. Data are shown as mean ± SD.

We next characterized anti-*Pneumocystis* IgMs in human cord blood from 9 human cord blood donors. We detected the presence of anti-*Pneumocystis* IgMs in all of the healthy cord blood samples, both by ELISA (Figure 2A) as well as by Western blotting in a subgroup of donors (based on sample availability) (Figure 2B, original unedited blots are depicted in Supplemental Figure 1; supplemental material available online with this article; https://doi.org/10.1172/jci.insight.161450DS1). Due to the multiple banding patterns shown by Western blotting, we next used cord blood from 3 human donors to perform Ig pull down assays and *Pneumocystis* proteomics as previously described (13). All 3 cord blood samples showed immunoprecipitation of PNEG_02182, a mitochondrial nuclease, and 2 of 3 samples showed immunoprecipitation of the peptidases PNEG_01105, PNEG_01848, and a putative cell adhesion protein PNEG_01454 (Figure 2C). We prioritized surface fungal proteins for further study, given that they are likely important targets of protective epitopes. Based on this, we prioritized PNEG_01454 as well as Meu10, a glycosylphosphatidylinositol-anchored protein, we had identified previously, thought to be involved in ascospore assembly (13). We produced these proteins in CHO cells and used these recombinant proteins for ELISA assays utilizing the other cord blood donor samples for validation. We found substantial IgM titers in independent cord blood samples (not used for immunoprecipitation) in response to both Meu10 and PNEG_01454 (Figure 2D).

### Adaptive immunity to PC Ags.

**Figure 3:**
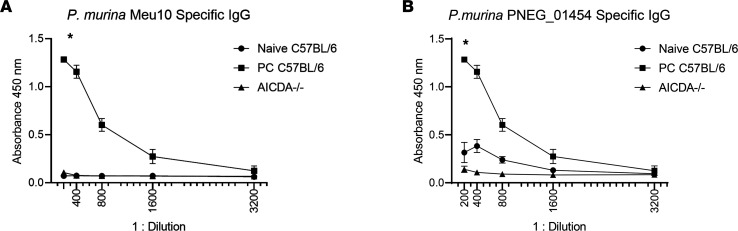
*Pneumocystis-*specific IgG responses 6 weeks after PC infection in mice. (**A**) *P*. *murina* Meu10/ PNEG_01435–specific IgG titers (*n* = 3 per group). Responses were only observed in PC-infected WT mice and not in naive C57BL/6 mice or *Aicda^–/–^* mice. (**B**) *P*. *murina* PNEG_01454–specific IgG titers (*n* = 3 per group). Two-way ANOVA analysis showed that IgG titers for each *Pneumocystis*-specific antigen were significant in C57BL/6 mice. *P < 0.05. Data are shown as mean ± SD.

We next determined whether PC-infected mice had adaptive IgG responses to these Ags. We infected C57BL/6 and *Aicda^–/–^* mice with *P*. *murina* and then assessed IgG responses to both Meu10 and PNEG_01454. Wild-type mice generated significant anti-Meu10 and PNEG_01454 IgG, which was associated with fungal clearance (Figure 3). In contrast, we observed no antifungal IgG in *Aicda^–/–^* or naive mice (Figure 3).

**Figure 4:**
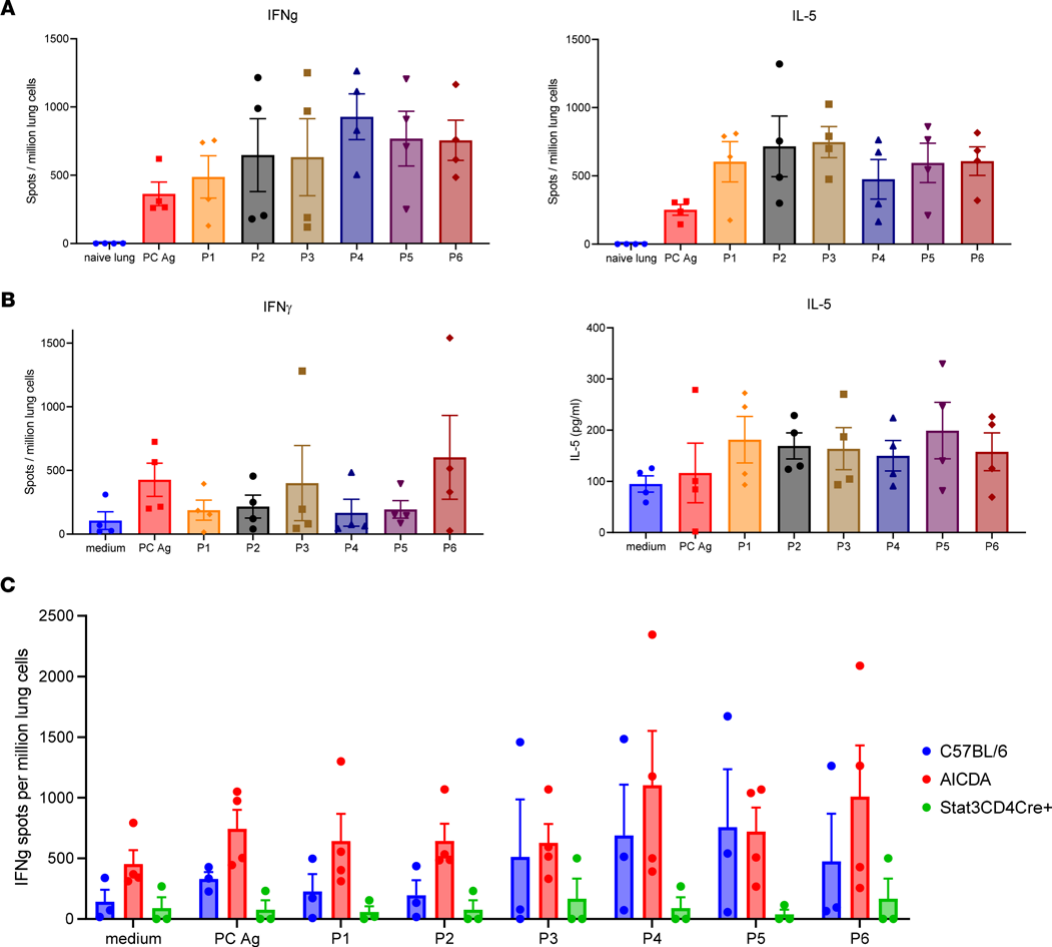
*Pneumocystis-*specific T cell responses after PC infection in mice. (**A**) C57BL/6 and (**B**) *Aicda^–/–^* mice were infected with 100 μL *P*. *murina* inoculum (2 × 10^5^ cysts) via oral pharyngeal administration. Two weeks later, single cells from mouse lungs were prepared for IFN-γ and IL-5 Elispot or LegendPlex assays (*n =* 4 per group). Uninfected naive lungs had a background of 0–1 spots in all stimulation conditions, so 1 representative naive lung sample is depicted as background in the assay. PC antigen and P1–P6 were significantly greater in lung samples from *Aicda^–/–^* mice compared with those from naive lung controls (*P* < 0.05 by ANOVA or Mann-Whitney test). (**C**) C57BL/6 *Aicda^–/–^* and CD4^+^ cre *Stat3^fl/fl^* mice at 6 weeks. Both C57BL/6 and *Aicda^–/–^* mice had significantly higher type I responses compared with CD4^+^ cre *Stat3^fl/fl^* mice (*P* < 0.05, ANOVA). Data are shown as mean ± SD.

Next, we queried T cell immunity in response to these Ags. For this, we performed whole-Ag stimulation of designed putative class II MHC–presented peptides for 2 of the Ags immunoprecipitated by human cord blood, PNEG_01454 and PNEG_02812. *Pneumocystis* induces a mixed T cell response, so for this study we assessed IFN-γ (type I) and IL-5 (type 2) Elispot responses at 2 weeks after inoculation, a time of peak T cell priming in the lung during infection. We designed 3 peptides for PNEG_01454 (P1–P3) and 3 for PNEG_02812 (P4–P6) (see Supplemental Figures 2 and 3). We detected substantial IFN-γ and IL-5 Elispot responses to these peptides in PC-infected C57BL/6 mice (Figure 4A). In contrast, cells from naive uninfected lung had 0–1 spots in these assays (Figure 4A). These IFN-γ and IL-5 responses were largely preserved in *Aicda^–/–^* mice (Figure 4B). Note that the medium controls have signal in these assays, because at the 2-week time point, there is significant PC Ag present in the lungs (14). We next assessed type I memory responses at 6 weeks after infection, when PC infection is resolved in WT mice. Again, we observed substantial type I responses to P1–P6 in both WT mice and *Aicda^–/–^* mice, denoting that T cell priming is preserved in *Aicda^–/–^* mice (Figure 4C). Notably, these responses were substantially reduced in CD4^+^ STAT3-deficient mice, demonstrating that these responses require STAT3 signaling T cells.

**Figure 5:**
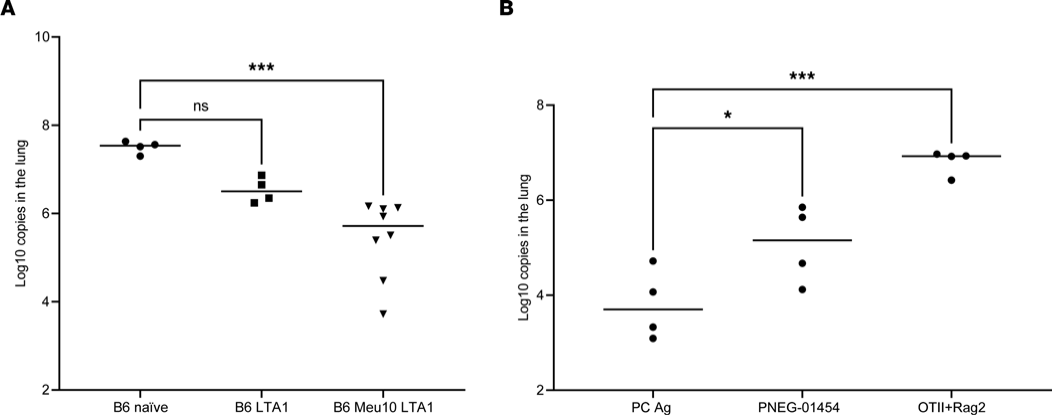
Effector activity of antigen-enriched CD4^+^ T cells. (**A**) C57BL/6 mice were immunized with 10 μg Meu10 and 10 μg LTA1, 10 μg LTA1 only, or vehicle (naive) by oral pharyngeal aspiration and boosted once 3 weeks later. One week after boosting, 2 × 10^5^ purified CD4^+^ T cells were transferred into *Rag2^–/–^* mice intravenously. Two weeks after cell adoptive transfer, all mice were infected with approximately 2 × 10^5^ asci of *P*. *murina* inoculum by oral pharyngeal aspiration. Four weeks later, right lung middle lobes were removed for RNA isolation and fungal burden assessment by RT-qPCR. Dunnett’s multiple-comparison test shows that *Rag2^–/–^* mice that received CD4^+^ T cells from Meu10-primed C57BL/6 mice had significantly lower fungal burden in the lungs compared with all other groups. (**B**) The experiments described in **A** were repeated with whole PC Ag and PNEG_01454. CD4^+^ T cells from OTII-*Rag2*^–/–^ mice were used as an irrelevant antigen control. *P < 0.05, ***P < 0.001 by 1-way ANOVA followed by Dunn’s multiple comparisons test.

To determine if these Ag-enriched CD4^+^ T cells had effector activity, we immunized mice with Meu10 and PNEG_01454 with the strong mucosal adjuvant LTA1, which induces robust Ag-specific Th1/Th17 responses (15). Vaccine-elicited cells were harvested from the lung and adoptively transferred into recipient *Rag2^–/–^* mice to allow homeostatic proliferation for 2 weeks followed by PC inoculation, as previously described (6). Fungal burdens were then assessed 4 weeks later. Animals containing cells elicited by Meu10 and LTA1 showed significantly reduced fungal burdens compared with those containing naive splenic CD4^+^ T cells or cells induced by LTA1 alone (Figure 5A). We next evaluated PNEG-01454. Again, CD4^+^ T cells from mice vaccinated with PNEG_01454 showed reduced fungal burden (Figure 5B) and nearly approached the effector activity of cells from mice immunized with whole PC Ag, whereas OTII cells with irrelevant Ag specificity had no antifungal activity (Figure 5B).

## Discussion

This study was based on prior studies that showed that B cells and *Pneumocystis*-specific B cell receptors are critical in priming pulmonary CD4^+^ T cell responses to this fungus (7–10). Although dendritic cells are thought to be critical in Ag presentation in the lung, B cells seem to be more critical for *Pneumocystis* infection. This may be due to the size of the ascus, as B cells have also been shown to be critical for Ag presentation of larger Ags, such as viral-like particles (16). Given the high incidence of *Pneumocystis* infection in children 2 years of age and under, but especially in infants (2, 3), we hypothesized that IgM-encoding B cell receptors appear critical in generating these early T cell responses. We have previously shown that IgM-deficient mice have delayed T cell priming when infected with *P*. *murina* (12).

To this end, anti-*Pneumocystis* IgM was readily detected in human cord blood as well as in naive mouse sera. Interestingly, samples from 3 of the human cord blood donors pulled down the same Ag in immunoprecipitation experiments. Using Ig-based proteomics, we were able to identify CD4^+^ T cell epitopes to *Pneumocystis*. We were able to express two of these antigens in CHO cells, and immunization with a strong CD4^+^ T cell adjuvant (15) elicited CD4^+^ T cells that showed effector activity in adoptive transfer experiments. Thus, human IgM may be very helpful in identifying CD4^+^ T fungal Ags that may aid in vaccination and also in diagnosis and determining if a patients have *Pneumocystis*-specific CD4^+^ T cells. This latter information may be useful in guiding *Pneumocystis pneumonia* prophylaxis. For example, an assay to detect *Pneumocystis*-specific T cells may be helpful in determining who is at greatest risk of *Pneumocystis pneumonia*. Future studies will be required to determine if the Ags identified here are also able to elicit human CD4^+^ T cell responses.

## Methods

### ELISAs.

Sera were obtained from WT C57BL/6, *Aicda^–/–^*, and *Rag2^–/–^* mice and assayed for whole anti-PC IgM by ELISA using PC Ag. Blood was collected at the time of sacrifice by syringe from the vena cave. Coagulated blood was then centrifuged for 10 minutes at 10,000*g*. The serum supernatant was collected and stored at –80°C. Maxisorb plates (Nunc/Thermo Fisher) were coated with 2 μg *P*. *murina* Ag in 100 μL of 1× sterile PBS per well overnight at 4°C. Plates were blocked with PBS with 1% Tween 20 and 10% BSA (Invitrogen, 88-50470). Plates were then stained with sample serum dilutions, covered, and incubated for 2 hours on a microplate shaker set at 400 rpm. Samples were aspirated and washed 4 times. Pretitrated HRP-conjugated anti-mouse Ig(H+L) polyclonal antibody (kit provided by Invitrogen, 88-50470) was added to all wells, covered, and incubated for 1 hour on a microplate shaker set at 400 rpm. Samples were aspirated and washed 4 times. 100 μL Tetramethylbenzidine substrate solution (Invitrogen, 88-50470) was added to each well and incubated at room temperature for 15 to 30 minutes, depending on control serum. The reaction was stopped with an equal volume of 2 N H_2_SO_4_. The optical density at 450 (OD_450_) was read using a BioTek Synergy HT microplate reader.

We screened cord blood donors (StemCell Technologies) using a similar ELISA method. We measured total IgM as follows. Total human IgM was measured using a IgM Human ELISA Kit with Plates (Thermo Fisher, Invitrogen, 88-50620) following the manufacturer’s protocol. Serum dilutions used for this ELISA were 1:100 and 1:1,000 in PBS with 1% Tween-20 and 10% BSA. For PC-specific IgM, Maxisorb plates were coated with 2 μg *P*. *murina* Ag in 100 μL of 1× sterile PBS per well overnight at 4°C. Plates were aspirated and washed twice. Plates were blocked with 200 μL of 2× PBS with 1% Tween 20 and 10% BSA (Invitrogen, 88-50470). Plates were aspirated and washed twice. Plates were then stained with sample serum dilutions, covered, and incubated for 2 hours at room temperature on a microplate shaker set at 400 rpm. Plates were aspirated and washed 4 times. Goat anti-human IgM (Southerbiotech, 2020-05) was diluted 1:4,000 in 1× PBS with 1% Tween-20 and 10% BSA, and 100 μL was added to all wells, covered, and incubated for 1 hour at room temperature on a microplate shaker set at 400 rpm. Plates were aspirated and washed 4 times. 100 μL Tetramethylbenzidine substrate solution (Invitrogen, 88-50470) was added to each well and incubated at room temperature for 15 to 30 minutes, depending on control serum. The reaction was stopped with an equal volume of 2 N H_2_SO_4_. OD_450_ was read using a BioTek Synergy HT microplate reader.

### Proteomics.

Samples from 3 human cord blood donors (0126, 0226, and 0091) were used for immunoprecipitation experiments. *Pneumocystis* protein Ags were isolated with the Pierce MS-compatible Magnetic IP Kit [Protein A/G] (Thermo Scientific, 90409) according to the manufacturer’s instruction. Briefly, 1 mg raw *Pneumocystis* material was prepared from *Pneumocystis*-infected *Rag2^–/–^IL2rg^–/–^* mouse lungs (13), and *Pneumocystis* was incubated with 0.5 mL human cord blood plasma overnight at 4°C. The immune complexes were incubated with MS-magnetic beads at room temperature for 1 hour with mixing. Then, the beads were collected, washed, and treated with IP-MS Elution buffer (a component in the Pierce MS-Compatible Magnetic IP Kit [Protein A/G]; Thermo Scientific, 90409). The elution containing the target Ags was further processed for MS analysis at Proteomics Core Facility in Louisiana State University Health Sciences Center at New Orleans.

### Peptide predictions and recombinant proteins.

Three peptides from the *Pneumocystis jirovecii* (strain RU7) acetylcholinesterase-domain-containing protein (A0A0W4ZWB9, PNEG_01454/T551_00157) and 3 peptides from the *P*. *jirovecii* mitochondrial endonuclease (A0A0W4ZRF7, PNEG_02812/T551_01500) were predicted to contain CD4^+^ T cell epitopes based on the highest combined scores of I-A^b^ bindings from NETMHCII (version 2.3) (17) and in-house calculation of the Ag processing likelihood (APL) (18). For the APL calculation, homology models were prepared using SWISS-MODEL (19), with PDB entries 6h14 and 6iid, respectively, as structure templates. Parameters for the APL calculation and the relative weight of I-Ab binding (60%) were obtained by optimization, with a benchmark set of CD4^+^ T cell–epitope maps reported for 12 Ags of known structure, representing 62 epitopes in C57BL/6 mice (20). Profiles of epitope prediction are presented in Supplemental Figure 2. Peptide sequences are included in Supplemental Figure 3. We produced recombinant Meu10 and PNEG_01454 in CHO cells (Curia, formerly LakePharma); however, we were unable to express recombinant PNEG_02812.

### In vivo T cell responses.

*Pneumocystis* was propagated in *Rag2/Il2rg^–/–^* mice (Taconic Biosciences) as previously described (6). To assess T cell responses, 6- to 10-week-old female C57BL/6 and *Aicda^–/–^* mice (both from The Jackson Laboratory) were infected with *Pneumocystis* as previously described (6, 21). Mice were euthanized at week 2 or week 6 for lung IFN-γ Elispsot assay (MabTech, product code 3321-2A) (13) as a measure of Th1 response or measurement of IL-5 in supernatant for type 2 responses using a LegendPlex assay (BioLegend). For the 6-week study, we included CD4-cre *Stat3^fl/fl^* mice as a negative control, because these mice fail to prime T cell responses to *Pneumocystis* (6).

### Immunization, cell purification, adoptive transfer, and Pneumocystis infection.

To generate *Pneumocystis* Ag–specific CD4^+^ T cells, female C57BL/6 mice were intratracheally immunized with 10 μg Meu10 plus 10 μg LTA1, 10 μg PNEG_01454 plus 10 μg LTA1, or 10 μg LTA1 only and boosted once 3 weeks later. We chose LTA1 because it induces strong lung Th1 and Th17 cells (15). One week after boosting, the lungs were harvested and single-cell suspensions were prepared. Lung CD4^+^ T cells were purified by using the EasySep MouseCD4^+^ T Cell Isolation Kit (StemCell Technologies, 19852). Control naive CD4^+^ T cells or OTII × *Rag2^–/–^* CD4^+^ T cells were purified from spleens. 2 × 10^5^ purified CD4^+^ T cells were adoptively transferred into Rag2 mice via retro-orbital injection and allowed to homeostatically proliferate as previously described (6). Two weeks after receiving CD4^+^ T cells, *Rag2^–/–^* mice were infected with *P*. *murina* (2 × 10^5^ cysts) via oral pharyngeal administration. Mice were euthanized 4 weeks later to assess fungal burden as previously described (6).

### Statistics.

All statistical analyses were performed using GraphPad Prism (version 9.2.0). Differences in antibody titers were analyzed using area under the curve analyses or comparison of the 1:32 OD by Mann-Whitney test. Elispot responses and fungal burdens were analyzed by 1-way or 2-way ANOVA followed by followed by Tukey’s or Dunn’s multiple comparisons test (within Prism). *P* values of less than 0.05 were considered significant.

### Study approval.

All animal experiments were approved by the Tulane IACUC under protocol ID 968. Human cord blood was purchased as deidentified samples and, thus, was IRB exempt.

## Author contributions

The work was conceived by KN, SJL, and JKK. KN, GD, DP, AE, JEM, SJL, and JKK designed and performed experiments and wrote the paper.

## Supplementary Material

Supplemental data

## Version 1 08/02/2022

In-Press Preview

## Version 2 09/08/2022

Electronic publication

## References

[1] Vera C, Rueda ZV (2021). Transmission and colonization of *Pneumocystis jirovecii*. J Fungi (Basel).

[2] Vargas SL (2013). Near-universal prevalence of *Pneumocystis* and associated increase in mucus in the lungs of infants with sudden unexpected death. Clin Infect Dis.

[3] Vargas SL (2001). Search for primary infection by *Pneumocystis*
*carinii* in a cohort of normal, healthy infants. Clin Infect Dis.

[4] Pneumonia Etiology Research for Child Health Study G (2019). Causes of severe pneumonia requiring hospital admission in children without HIV infection from Africa and Asia: the PERCH multi-country case-control study. Lancet.

[5] Ochoa S (2020). Genetic susceptibility to fungal infection in children. Curr Opin Pediatr.

[6] Elsegeiny W (2018). Murine models of *Pneumocystis* infection recapitulate human primary immune disorders. JCI Insight.

[7] Elsegeiny W (2015). Anti-CD20 antibody therapy and susceptibility to *Pneumocystis* pneumonia. Infect Immun.

[8] Lund FE (2003). Clearance of *Pneumocystis*
*carinii* in mice is dependent on B cells but not on P carinii-specific antibody. J Immunol.

[9] Lund FE (2006). B cells are required for generation of protective effector and memory CD4 cells in response to *Pneumocystis* lung infection. J Immunol.

[10] Opata MM (2015). B lymphocytes are required during the early priming of CD4+ T cells for clearance of *Pneumocystis* infection in mice. J Immunol.

[11] Rapaka RR (2019). CD4^+^ T cell regulation of antibodies cross-reactive with fungal cell wall-associated carbohydrates after *Pneumocystis murina* infection. Infect Immun.

[12] Rapaka RR (2010). Conserved natural IgM antibodies mediate innate and adaptive immunity against the opportunistic fungus *Pneumocystis murina*. J Exp Med.

[13] Zheng M (2014). Novel *Pneumocystis* antigen discovery using fungal surface proteomics. Infect Immun.

[14] Beck JM (1991). Reduction in intensity of *Pneumocystis*
*carinii* pneumonia in mice by aerosol administration of interferon-gamma. Infect Immun.

[15] Iwanaga N (2021). Vaccine-driven lung TRM cells provide immunity against *Klebsiella* via fibroblast IL-17R signaling. Sci Immunol.

[16] Hong S (2018). B cells are the dominant antigen-presenting cells that activate naive CD4^+^ T cells upon immunization with a virus-derived nanoparticle antigen. Immunity.

[17] Jensen KK (2018). Improved methods for predicting peptide binding affinity to MHC class II molecules. Immunology.

[18] Mettu RR (2016). CD4+ T-cell epitope prediction using antigen processing constraints. J Immunol Methods.

[19] Waterhouse A (2018). SWISS-MODEL: homology modelling of protein structures and complexes. Nucleic Acids Res.

[20] Charles T (2022). CD4+ T-Cell Epitope Prediction by Combined Analysis of Antigen Conformational Flexibility and Peptide-MHCII Binding Affinity. Biochemistry.

[21] Eddens T (2019). Transcriptomic and proteomic approaches to finding novel diagnostic and immunogenic candidates in *Pneumocystis*. mSphere.

