# Supplementary material for: Germline IgM predicts T cell immunity to Pneumocystis
Source: JCI Insight. 2022 Sep 8;7(17):e161450. doi: 10.1172/jci.insight.161450 (PMC9536272; doi:10.1172/jci.insight.161450)
Supplement: Supplemental data [file jciinsight-7-161450-s158.pdf]

Supplement Figures

Figure S1. Original Western Blots.

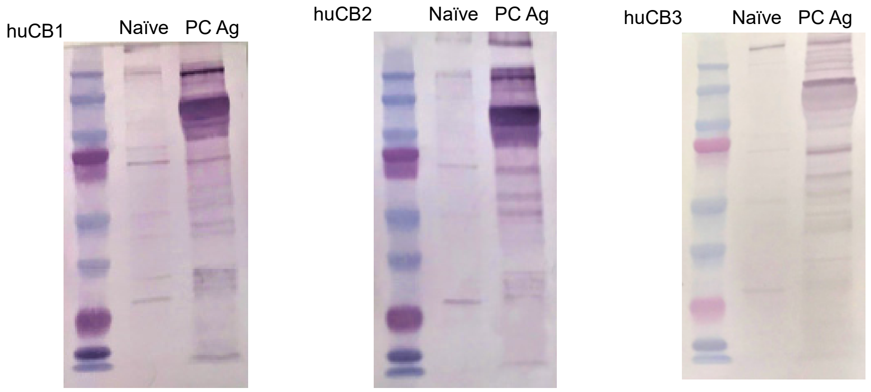

STEMCELL Technologies		
Human CB Plasma		
huCB1	Cat# 70020.2	Lot# 1903110126
huCB2	Cat# 70020.2	Lot# 1903190226
huCB3	Cat# 70020.1	Lot# 1810100091

Naïve *Rag2-/-Il2rg-/-* lung preparation and PC antigen were run on the 10% SDS-PAGE gel and transferred to PVDF membrane. Membranes were cut and incubated with human CB plasma samples as outlined: donors 0126, 0226, 0091. The membrane was then probed with goat anti-human IgM-AP (Invitrogen A18838) and subtract. This process was repeated with the additional donors 4011, 5030, 0018, and 9020 below. In this blot PC antigen is the lane immediately to the right of the marker.

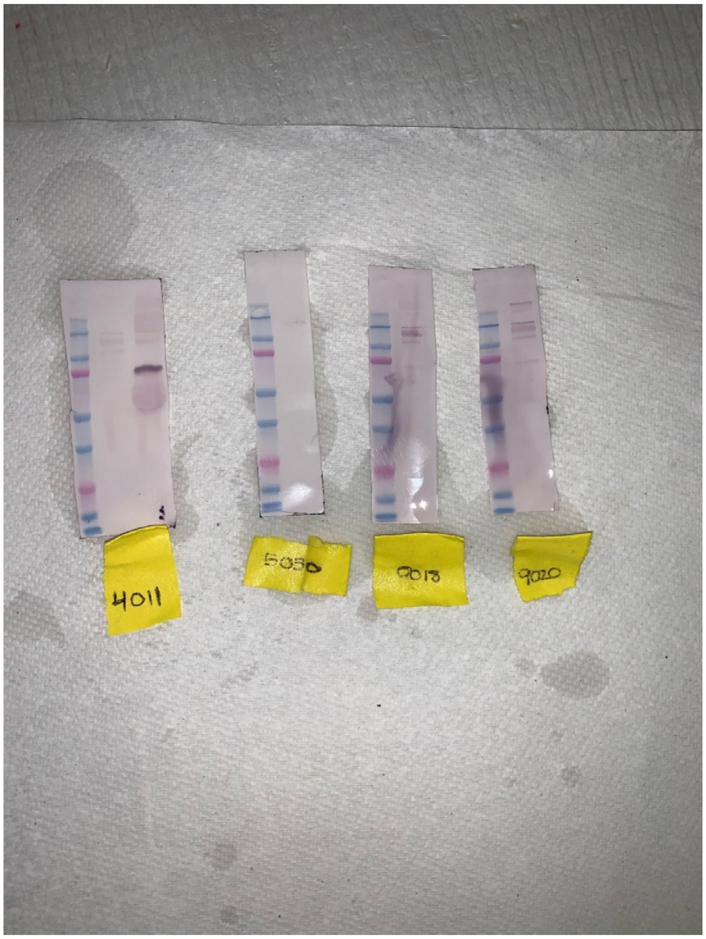

Figure S2.

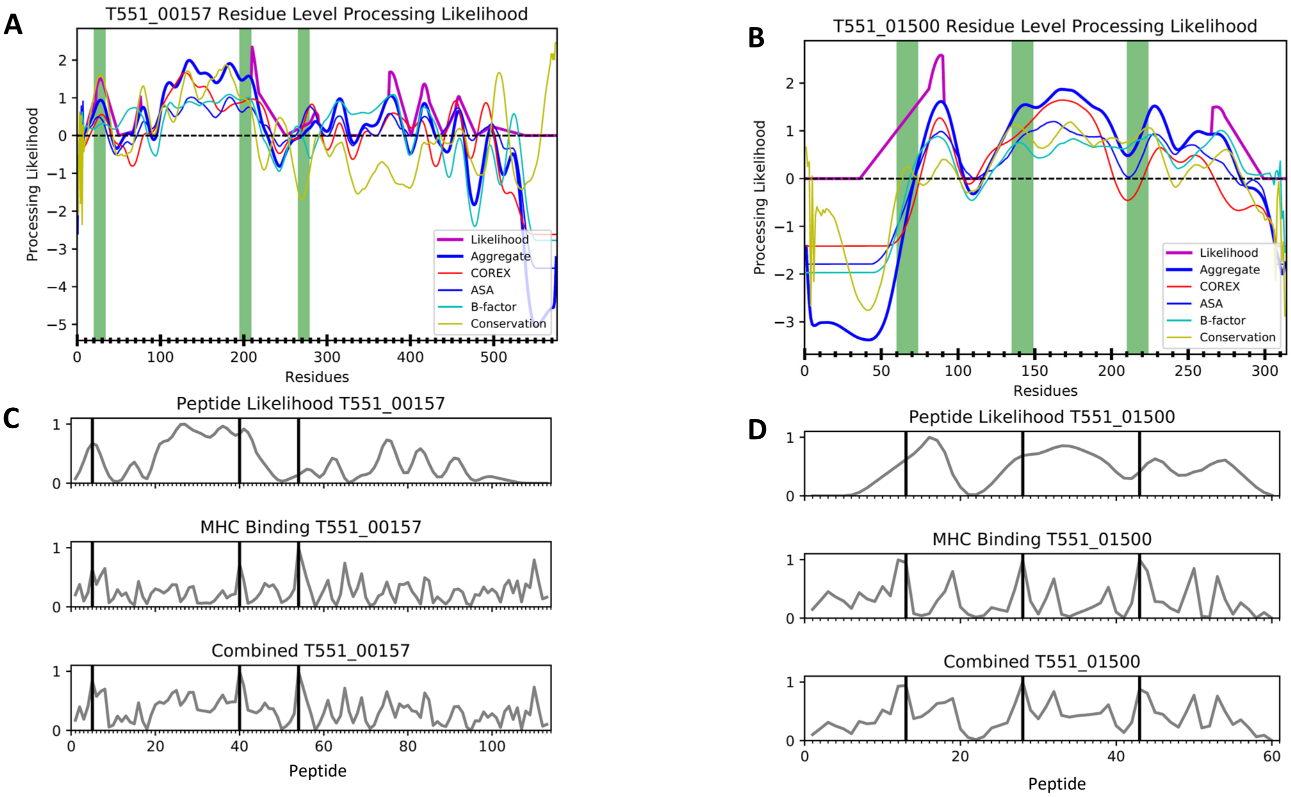

Figure S2. CD4+ T-cell epitope prediction using a combination of Antigen Processing Likelihood and class II MHC (I-A^b) binding. Conformational stability and residue-level processing likelihood for *Pneumocystis jirovecii* (strain RU7) acetylcholinesterase-domain containing protein, PNEG_01454/T551_00157 (A) and mitochondrial nuclease, PNEG_02812/T551_01500 (B) were calculated as described in PMID: 29315598. Peptide-level processing likelihood, MHCII binding, and combined profiles (C,D) illustrate the average values for the overlapping series of 15-mer peptides in 5-residue steps. Vertical lines indicate the top-three peptides in combined epitope-prediction score.

Figure S3.

Peptide	Sequence	within	AA identity *jiroveci/murina*
P1	FLFLGIPYAEPPVGK	Cell adhesion protein PNEG_01454/T551_00157	12 of 14
P2	AKELFQGAVSASDPI	Cell adhesion protein PNEG_01454/T551_00157	9 of 12
P3	GGIFTSFQTSSITAA	Cell adhesion protein PNEG_01454/T551_00157	8 of 10
P4	LYQYSNPRPISDCLV	Mitochondrial Nuclease PNEG_02812/T551_01500	Mitochondrial Nuclease PNEG_02812
P5	YDRGHLAPAANAKFS	Mitochondrial Nuclease PNEG_02812/T551_01500	10 of 15
P6	WRVSYEMIGSPPNVA	Mitochondrial Nuclease PNEG_02812/T551_01500	13 of 15

Peptides were manufactured by Alan Scientific Inc.
